# How entomological studies can help the control of mosquito-borne diseases: a five-years experience in north-eastern Italy

**DOI:** 10.1186/1756-3305-7-S1-O30

**Published:** 2014-04-01

**Authors:** G Capelli, F Montarsi, S Ravagnan, S Cazzin, M Mazzucato, P Mulatti, F Russo, S Marangon

**Affiliations:** 1 Istituto Zooprofilattico Sperimentale delle Venezie, Legnaro (Padua), Italy; 2 Direzione Prevenzione, Veneto Region, Venice, Italy

## 

North-eastern Italy is particularly suitable for mosquito survival, due to its climate, landscape and abundance of wild/domestic animals. After the emergence of West Nile Virus (WNV) in 2008, entomological studies were implemented. Here we describe how entomological data were managed to optimize surveillance and control of mosquito-borne pathogens.

CDC-CO_2 _traps were used (May-October, 2009-2013). In one site, captures with CDC and gravid traps were organized every 2hrs for 24hrs. In other three sites pre- and post-disinfestation captures were done. The mosquitoes were screened by RT-PCR for Flaviviridae and a sub-sample for Bunyaviridae. Host preference of *Culex pipiens *was assessed by PCR blood meal analysis. Mapping, modelling and spatial analyses were done using entomological data to identify correlations with climate, landscape, animal and human infections.

More than 700,000 mosquitoes were collected, with *Cx.pipiens *the most abundant (80%) and the only vector of WNV and USUV. Tahyna virus was isolated once in *Ochlerotatus caspius*. *Cx.pipiens *fed preferentially on birds (76%), mainly blackbird, sparrow, magpie and collared dove, but not on humans. Diel activity showed that *Cx.pipiens *changed its host searching activity according to the season and defined the favorite period for oviposition. The success of disinfestation measures in reducing *Cx.pipiens *density varied according to the methods used. The contribution of density-dependence in regulating vector population growth resulted greater than any environmental factor on its own. Overall the most significant predictors of *Cx.pipiens *dynamics included length of daylight, population density and temperature in the 15 days prior to sampling. Precipitation, number of rainy days and humidity had limited importance. Linear models detected significant relationships between WNV in humans and mosquitoes. Spatial analysis detected clusters of WNV occurrences for all the hosts, identifying an area where to focus surveillance and promptly detect WNV re-activation.

In long term studies the mosquito species composition, seasonality, distribution, abundance and pathogen rate of infections were assessed. The blood-meal analyses indicated possible bird targets for surveillance. The control of the efficacy of disinfestations highlighted the need for harmonic guidelines. Modelling indicated the need to incorporate density dependence in combination with key environmental factors for robust prediction of *Cx.pipiens *population expansion, helping to identify when and where an increase in vector population size and WNV transmission risk should be expected. The results stressed also the necessity to improve the density and the frequency of mosquito captures.

## Funding

Veneto and Friuli Venezia Giulia regions, Italian Ministry of Health.

